# Chemometric approach to evaluate the chemical behavior of rainwater at high altitude in Shaune Garang catchment, Western Himalaya

**DOI:** 10.1038/s41598-022-15422-0

**Published:** 2022-07-27

**Authors:** Ramesh Kumar, Rajesh Kumar, Atar Singh, Mohammad Arif, Pankaj Kumar, Anupma Kumari

**Affiliations:** 1 Department of Environmental Science, School of Earth Sciences, Central University of Rajasthan, Bandar Sindri, Ajmer, Rajasthan India; 2 National Institute of Urban Affairs, Ministry of Housing and Urban Affairs, Delhi, India; 3 Integrated Regional Office, Ministry of Environment, Forest and Climate Change (MoEFCC), Government of India, Saifabad, Hyderabad, Telangana India; 4 Environmental Biology Laboratory, Department of Zoology, Patna University, Patna, Bihar, India

**Keywords:** Environmental sciences, Environmental chemistry, Atmospheric chemistry

## Abstract

The present research has been performed to analyze the chemical behavior of rainwater of the Shaune Garang catchment (32.19° N, 78.20° E) in the Baspa basin, located at a high elevation (4221 m above mean sea level) in the Himachal Himalaya, India. During the study period, sixteen rainwater samples were collected from the Shaune Garang catchment at five different sites. The volume-weighted mean (VWM) pH value of rainwater ranged between 4.59 and 6.73, with an average value of 5.47 ± 0.69, indicating the alkaline nature of rainfall. The total ionic strength in the rainwater ranged from 113.4 to 263.3 µeq/l with an average value of 169.1 ± 40.4 µeq/l. The major dominant cations were Ca^2+^ (43.10%) and Na^+^ (31.97%) and anions were Cl^−^ (37.68%), SO_4_^2−^ (28.71%) and NO_3_^−^ (23.85%) in rainwater. The ionic ratios were calculated among all the ions. The fraction of (NO_3_^−^  +Cl^−^) with SO_4_^2−^ was measured as 2.3, which specifies sour faces of rainwater due to HNO_3_, H_2_SO_4_, and HCl. A multivariate statistical assessment of rainwater chemistry through Principal Component Analysis (PCA) shows the significance of four factors controlling 78.37% of the total variance, including four-component (PC1 explained 27.89%, PC2 explained 24.98%, PC3 explained 14.64%, PC4 explained 10.85%). However, the individual contribution of Factor 1(PC1) explains 27.89% of the total variance (78.37%) and displays a strong optimistic loading for Ca^2+^ and Cl^−^. Further, high loading of Ca^2+^ and NO_3_^−^ and moderate loading of SO_4_^2−^ signify the contribution of burning fossil fuel and soil dust. Anthropogenic and natural pollutants influence the composition of rainwater in the pristine Himalayas due to local and long-distance transportation. The study area receives precipitation from the West and North-West, transporting dust and fossil fuel emissions from the Thar Desert and Northwestern countries.

## Introduction

The Himalayan region provides a unique ecosystem and water resource for many rivers in countries like India, Nepal, Bhutan, Pakistan, and China. The Himalayan glaciers serve as the "water towers" and provide significant meltwater to the downstream population^1^. However, the retreat of the Himalayan glaciers due to alteration and variation in the precipitation pattern in the regime impacts climate change^2^. Also, atmospheric pollution such as aerosol, dust, and particulate matter threatens the Himalayan region by altering the radiation budget. In recent years, atmospheric pollutants have increased to an extreme degree due to the lucrative growth in population. The increased population induces an increase in industrialization, urbanization, and energy consumption^3–5^. In addition, air contamination is the fifth most prominent threat to death globally, larger than food scarcity, alcohol consumption, and a physically inactive lifestyle^6^. Rainwater chemistry studies reflect the air contamination provided to the atmosphere by natural or anthropogenic sources^6^. It aids in determining the comparative significance of various causes and forecasting likely acidification buffering ability in the future^7^. It also aids in the understanding of the quantification of pollution removed from the atmosphere. As a pollutant removal mechanism, atmospheric precipitation (such as rain, dew, and snow) is the most effective. It can remove a wide range of chemical species and atmospheric aerosols, soluble gases, and the contribution of various pollutant sources (crustal, marine, anthropogenic, and natural)^8–13^. According to studies, rainwater acidity has become a significant environmental hazard, affecting soil texture, groundwater quality, vegetation, plants, and human health. As a result, World Meteorological Organization established the Global Atmospheric Watch program to monitor changing rainwater chemistry worldwide through several station networks.

In addition, the chemical configuration of rainwater is directly related to different factors such as native radiations, contaminant transportation, climatic situations, and the size of the drop. Atmospheric particles play a critical role in forming clouds and rain and influence energy distribution in the atmosphere. The particles emitted by the natural and anthropogenic sources can travel long distances from the origin and be sifted by different atmospheric mechanisms^14,15^. The presence of particles in the atmosphere is determined by various mechanisms, including the washout process known as below-cloud scavenging and in-cloud scavenging known as rainout. The washout process removes coarse mode particles from the atmosphere, whereas the rainout practice integrates fine-mode particles^16,17^. The climatic impact of the atmospheric particles is not well understood due to the lack of documented study regarding rainwater's physical and chemical composition^18^. Previous research on rainwater chemistry has been done to determine the physio-chemical composition and its possible sources in several parts of the world, including India^15,19–22^, but such studies are rare in the Himalayan region.

Hence, rainwater chemistry is essential to understanding the mechanism of pH and EC fluctuation at greater altitudes in the Himalayan region. Significant research has been conducted in the Himalayan region to determine rainwater chemistry^23–26^. Therefore, the primary goal of the present investigation is to define the chemical properties of rainwater and assess the impact of anthropogenic and natural emissions sources in rainwater. Furthermore, statistical techniques and trajectory analysis are performed to classify potential causes of rainwater composition. Besides these, probably no documented works are available on rainwater chemistry in the high altitude in Sangla Valley Baspa basin. Therefore, we have focused on a detailed study to fill the research gap covering physical and chemical characteristics and possible nutrient sources. In addition, this study could be significant for a better understanding of the role of the weathering and hydrological process in the catchment. A particular focus has been made on the multivariate statistical assessment of rainwater chemistry.

### Study area

The research area, Shaune Garang glaciated basin, is located between 31° 16′ 45" N and 31° 18′ N and 78° 18′ 30" E and 78° 22′ E in the Baspa Basin, Himachal Himalaya. Meltwater from the Shaune Garang glacier flows into the Baspa River at Sangla. The catchment's total area is approximately 60 km^2^, while the area above the discharge gauging site is 38.13 km^2^ ^27–30^. Climatic parameters are the primary regulators of the mountain ecosystem and glacier dynamics. Due to higher temperatures, the area is subjected to ablation from May to September^27^. This catchment receives precipitation from the monsoon in summer^31^ and westerlies in winter through Western Disturbance (WD)^32^. An excessive inconsistency in rain and snowfall patterns has been reported in the Himalayas, with varying amounts of precipitation of 100 to > 1600 cm^33^, depending on the terrain and local climatic conditions. The contribution of the monsoon is more considerable, mainly in the eastern Himalayas, and reduces towards the western Himalayas.

**Figure 1:**
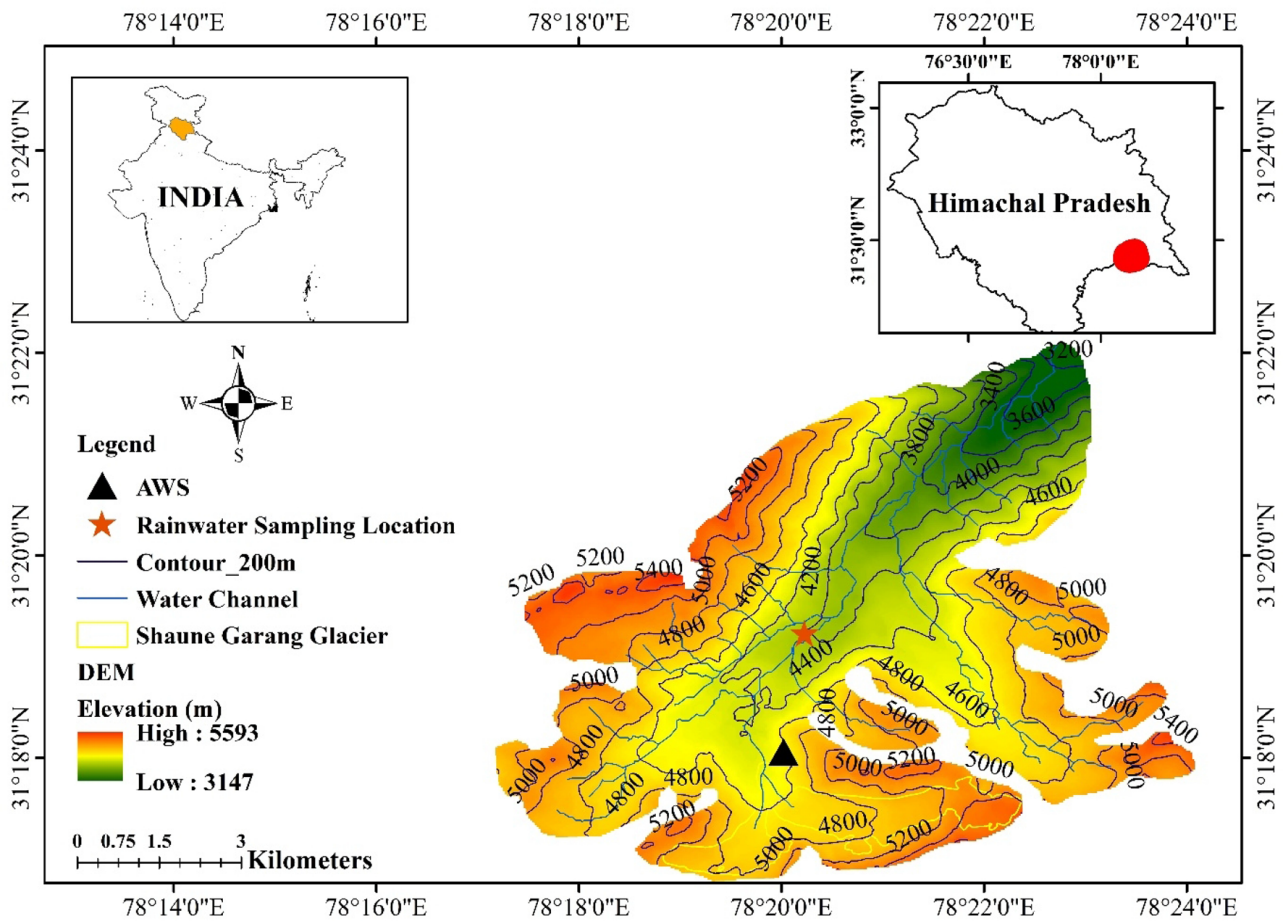
Map shows the rainwater sampling site and automatic weather station (AWS) at high altitudes in Shaune Garang, Himachal Himalaya. The study area map is created using a geographical information system in the DST-FIST-funded GIS Laboratory, Department of Environmental Science, Central University of Rajasthan (ArcGIS 10.1; version 10.1 and authorization number: EFL691568009-1010). Freely available data on India shapefile (http://www.diva-gis.org/gdata), Digital Elevation Model (DEM) at NASA Earth Data (https://search.earthdata.nasa.gov/search/) were used.

In contrast, the westerly contributes more to the western parts of the Himalayas and declines towards the eastern. The rocks of this region resemble the Higher Himalayan Crystalline. It contains pelitic and psammopelitic meta-sediments having acidic and basic intrusive. Different types of granite and gneiss rocks are found in the Himalayan region and have an ordinary presence of late-stage pegmatitic veins. This also has light grey-green coloured feldspar. Rohtang gneiss is the principal constituent of this basin. Chalcopyrite is also obtained in lateral morainic deposits of the Himalayas. The study area has major geologic components of the "Rakcham group of granite"^34^. The location map presented in Fig. 1 shows the rainwater sampling site and automatic weather station (AWS) at high altitudes in Shaune Garang catchment, Himachal Himalaya.

### Water sample collection and chemical analysis

The ionic study of cations and anions in rainwater was performed in the Shaune Garang catchment during the ablation season (June to September) in 2017. Sixteen samples of rainwater were collected throughout the study period in the catchment. Rainwater samples were collected in a rain collector made of a polyethylene bucket at elevations ranging from 3500 to 4500 m asl. The sample sites were divided into five zones in the catchment. Before collecting rainwater samples, the bucket was cleansed and rinsed twice with distilled water. Rainwater samples were filtered using 0.45 µm filter paper into a prewashed, cleaned polypropylene bottle. The samples were collected event-wise during the study period. As such, rain events do not have any human control or bias. So, they can be considered random events, and the sampling of rainwater events was "random sampling." Collections were done using the manual bucket method with a high-density polypropylene bucket. The collections were done at the height of 5 m above ground to prevent contamination of samples from splashes.

The pH and EC were observed on-site by a handheld multi-parameter instrument (HANNA) before the samples were calibrated with standard buffer solutions. The handheld multi-parameter instrument (HANA, model number HI9829) is a waterproof portable logging multi-parameter. The multi-sensor probe is based on a microprocessor, making it possible to measure critical parameters such as pH, ORP, conductivity, dissolved oxygen, and turbidity. Rainwater samples were kept at 4 °C until analysis. Atomic Absorption Spectroscopy was used to determine the essential cations (Ca^2+^, Mg^2+^, K^+^, Na^+^). For Ca^2+^, Mg^2+^, K^+^, and Na^+^, the instrument has a detection limit of 0.05 ppm. Ion Chromatograph (PERKIN ELMEWR), Dionex ICS 900, USA, with a detection limit of 0.01 ppm, was used to analyze the anions (Cl^−^, SO_4_^2−^, and NO_3_^−^) at 0.25 ml/min eluent flow rate. We followed the sample analysis procedures adopted by Bisht et al. (2017), which incorporates the analysis of anions (Cl^−^, SO_4_^2−^, and NO_3_^−^), with a CO_3_^2–^/HCO_3_^–^ buffer serving as the eluent (1.7 mM Na_2_CO_3_/1.8 mM NaHCO_3_) for the isocratic analysis, and 25 mM H_2_SO_4_ serving as the reagent^35^. To prepare the stock solutions, sodium salts of each ion were diluted to a concentration of 100 ppm. The peak areas of the standards were used as a basis for the calculations used to determine the concentrations. The peak response was checked once every five samples by running standard triplicates. If the deviation was greater than two percent, then recalibration was done. The methodical accuracy was maintained using known standard solutions of ionic radicals. The NOAA Air Resources Laboratory's Hybrid Single-Particle Lagrangian Integrated Trajectory (HYSPLIT) model (http://www.arl.noaa.gov/ready/hysplit4.html) was used to access the origin of air parcels reaching an altitude of 1000 m in the Shaune Garang catchment. The rainfall data were obtained from the Climate Hazards center InfraRed Precipitation with Station data (CHIRPS). CHIRPS incorporates 0.05° resolution satellite imagery with in-situ station data to create gridded rainfall time series of 30 + year quasi-global data for trend analysis and seasonal drought monitoring.

## Result and discussion

### Data screening and quality control

**Figure 2:**
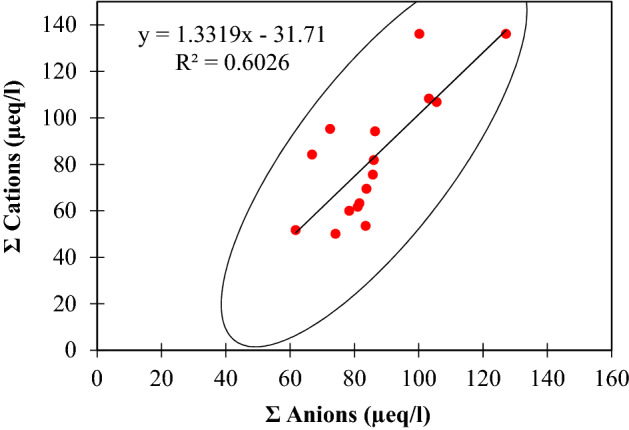
Correlation among the sum of anions and sum of cations during the ablation period of 2017.

The present investigation defines the chemical properties of rainwater to assess the impact of anthropogenic and natural emissions sources in rainwater in the higher Himalaya. This kind of research is scarce in the Himalayan region. Harsh terrain and inclement weather conditions are hurdles for the researchers in collecting the data and maintaining the quality. The sampling protocol has been followed with the utmost care from the sampling to preserving rainwater until the chemical analysis. In addition, the rainwater samples with any trace, filthy, messy, or contaminated with dust have been discarded. The data acquired through the chemical analysis were tested through the ion balance method. Data were examined to ensure analytical quality, considering calibration and blank dimensions. The electro-neutrality principle describes the preeminence of chemical analysis, which can be measured by the ionic balance in specific occurrences in samples. The ratio of cations to anions in rainwater samples ranged from 0.74 to 1.56, with an average value of 1.10 ± 0.24. The linear regression analysis helped determine the net charge balance between the cations (NH_4_^+^, Na^+^, K^+^, Ca^2+^, and Mg^2+^) and anions (Cl^−^, NO_3_^−^, SO_4_^2−^, and HCO_3_^−^) in the rainwater samples. This served as a quality control measure for the data. A linear regression of total cation against total anion has been performed to ascertain the data quality further. A significant linear regression (r = 0.60) between the total amount of anions and cations is observed in the samples (Fig. 2). The slope (m = 1.3) obtained from the linear regression deviated to the higher side of the ideal 1:1 reference line. The outcome designates that data quality is precise enough for further analysis. In the current investigation, the deviation of ≤ 40.4 for the total ion concentration of 169.14 µeq/l is within the acceptable range of 113.45–263.33 µeq/l. However, for high altitude samples, such ion-balance deviations may be caused by fluctuations in lower and higher ion concentrations and the presence of weak organic acids. A prevalent organic acid in the atmosphere, coupled with the H^+^ ion, is likely to evaporate fast in non-preserved water samples and their quantities were lower in rainwater samples^21^. The volume-weighted mean (VWM) concentrations of the observed chemical species in the Shaune Garang catchment rainwater were calculated as follows^36^:

1 $${\text{VWM}} \left( {\frac{{\upmu {\text{eq}}}}{l}} \right) = \frac{{\mathop \sum \nolimits_{i = 0}^{n} Ci Pi}}{{\mathop \sum \nolimits_{i = 0}^{n} Pi}}$$

Ci denotes the concentration of a specific chemical species (µeq/l). In contrast, Pi and N represent the quantity of rainfall for each event (in mm) and the total number of rainfall events, respectively.

### pH and EC variability in rainwater

**Figure 3:**
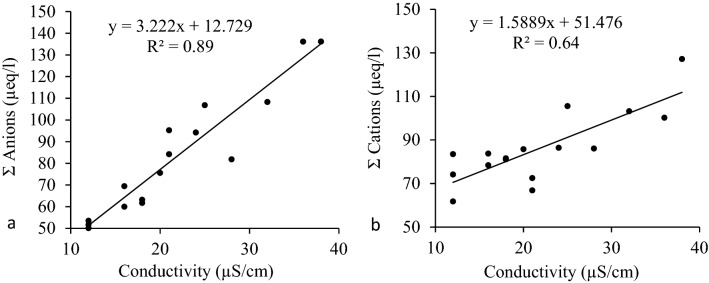
Scatter plot of conductivity against (**a**) sum of anions and (**b**) sum of cations.

The pH value of rainwater indicates the presence of major ionic constituents, either from atmospheric gas assimilation or anthropogenic sources. In the atmosphere, significant ionic species in rainwater are caused by in-cloud and sub-cloud scavenging processes^37^. Even though sulfuric and nitric acid is formed from nitrogen and sulfur oxides, they freely dissolve in cloud water and produce extra hydrogen ions. Furthermore, higher carbon dioxide levels (CO_2_) in the atmosphere dissolve in cloud water and produce weak carbonic acid^26^ (pH < 5.6, i.e., atmospheric CO_2_ balance), indicating the presence of acidic species in the ionic composition of rainwater. In contrast, a pH value of more than 5.6 specifies the existence of basic species, particularly crustal species or mineral dust^38^. The pH of rainwater fluctuated from 4.59 to 6.73. The mean value with the standard deviation of 5.47 ± 0.69 during the study period indicates a mixture of anthropogenic and natural chemical constituents in the catchment (Fig. S1a). The lowest pH value recorded was 4.59 on September 3, 2017, with 7.28 mm of rainfall, indicating that rainwater in the watershed is acidic on an occasional basis. On September 13, 2017, a high pH value was recorded along with 36.25 mm of rainfall and the presence of significant alkaline aerosols. Several studies at high altitudes in India and around the world report the average pH value of rainwater as 5.10–6.40. The variation in the pH and EC values of rain events during the study period over the sampling site is presented in Fig. S1b. In the Himalayas, at Kullu, pH value of rainwater was reported to range from 5.16 to 3.36^24^, while at Darjeeling, it varied from 5.0 ± 0.8^25^. The VWM pH of the rainwater was observed as 5.56 ± 0.29, indicating the mixture of anthropogenic and natural chemical constituents in the rainwater of Shaune Garang catchment. In addition, the other fundamental constituent of rainwater is the specific conductivity, which is used to check the quality of rainwater. The average specific conductivity observed in this valley was 13 to 36 μS/cm, with an average value of 22.31 ± 7.3. The specific conductivity in this catchment is lower than the reported value of 7 to 57 μS/cm in the Himalayan region^39,40^. The specific conductivity against the sum of anions and cations has been shown in Fig. 3. The findings show a stronger correlation with anions (R^2^ = 0.89) than with cations (R^2^ = 0.60). The high correlation representation by anion with the conductivity is due to the lower standard deviation of the data points.

For determining the rain acidity, linear regression analysis between the ionic concentration of hydrogen ion (H^+^) in rainwater and rainfall amount is plotted (Fig. S2). The correlation between the rainfall and Hydrogen ion concentration was found (r = 0.27) to be not well correlated. The linear regression slope (m = 0.12), upper side from the 1:1 equiline. The buffering capacity of rainwater could explain the lower pH levels observed over several hours of continuous rain. When comparing natural precipitation in equilibrium with atmospheric CO_2_ (pH = 6.73 and [H^+^] = 1.48µeq/1) to the lowest pH value of 4.59 throughout the study period, the difference in free acidity of [H^+^] is 3.62 µeq/1.

**Figure 4:**
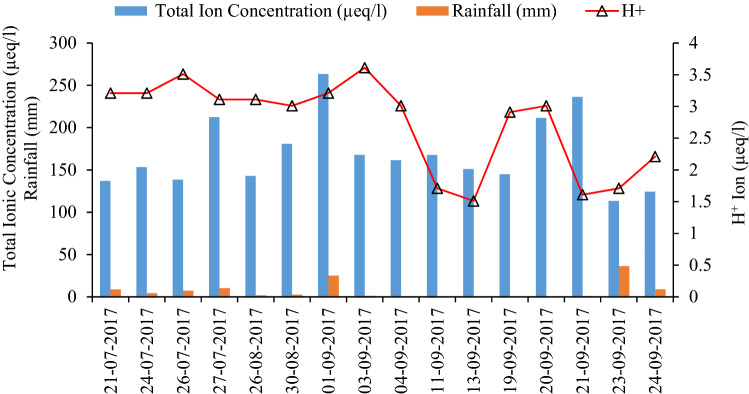
Average seasonal H^+^ ion variations, rainfall amount, and ion concentrations.

Furthermore, the highest pH of 6.73 (H^+^  = 1.48 µeq/l) correlates with a small concentration of hydrogen ions (H^+^  = 0.4) in 0.04 mm of rainfall in the Shaune Garang catchment (Fig. 4). The significant rain in the middle of the sampling period was a substantial factor in the dilution of hydrogen ions. Seasonal variations in rainfall amount and air quality have an impact on these.

### Chemical constituents of rainwater

A rigorous statistical analysis to determine the mean, volume-weighted mean (VWM), minimum, maximum, standard deviation, and standard error for each ion and pH has been provided in Table S1. The charge balance error (CBE) analysis of the chemical composition of dissolved ions in rainwater demonstrated data accuracy. As a result, the following empirical formula was used to calculate CBE:

2 $${\text{CBE}} = \frac{{\left( {{\text{TZ }}^{ + } - {\text{TZ}}.^{ - } } \right)}}{{\left( {{\text{TZ }}^{ + } + {\text{TZ}}.^{ - } } \right)}} \times 100$$

where TZ^+^  = Total cation, TZ^−^ = Total anion.

During the study period, the error in charge balance between total cation (TZ^+^) and total anion (TZ^−^) was found to be (6%), indicating that the dataset is more accurate. Table S1 summarizes the chemical composition of rainwater samples collected from the Shaune Garang glacier during the 2017 study period. There are very few studies of this nature in the Himalayas. However, we compared the VWM of rainwater composition with the analysis at Nainital (373.42 µeq/l)^26^ nearby the study area. The volume-weighted mean (VWM) of rainwater ionic composition was 187.75 µeq/l at Shaune Garang is substantially lower, indicating low atmospheric pollution concentrations in the Shaune Garang catchment. Looking at the tourism pressure and vehicular influence in the Himalayas, Nanital and Kullu are the hot spots. At the same time, the Shaune Garang region is less influenced by the tourism and may be the reason for the lower VWM. In addition, the altitude of Shaune Garang (4221 m asl) is much higher than Nainital (1958 m asl), making lower transportation of pollutants. It is further noted that the land use pattern is different at these three locations as well as the agricultural practices and population density is substantially lower at Shaune Garang, impacting less in terms of pollution with mean cations and anions were 85.33 µeq/l, and 80.11 µeq/l. The outcome demonstrates the supremacy of cations over anions in the catchment. The mean values of ionic species concentrations (167.63 µeq/l) are higher than the median values (157.32 µeq/l), indicating an irregular dispersal of ionic species with skewness to the left. The median and average concentrations show an analogous configuration. Therefore, VWM is used to recognize the greater concentrations during brief periods of rain and evade clean rain diluting and influencing the rainwater concentration^41^. The VWM concentration of Ca^2+^ was dominant among the cations in the study period, whereas K^+^ ion concentration was least dominant. The dominance of Ca^2+^ ions indicates the influence of mineral dust on rainwater chemistry. Being a marker of biomass burning, the occurrence of K^+^ ion indicated the prevalence of biomass burning in the region^20,42^. Ca^2+^ and Mg^2+^ in precipitation show the influence of terrestrial sources, such as the dissolution of dolomites and limestones^10–12,41^. The presence of calcium can also be from anthropogenic activities, such as open quarries, and cement factories, while magnesium can be from marine sources. Among all ionic components, calcium contributed maximum (22.35%), followed by chloride (17.72%), sodium (16.39%), sulfate (13.62%), nitrate (11.81%), magnesium (8.97%), bicarbonate (5.06%) and potassium (4.08%). The acidic nature of water is generally due to the presence of sulfuric and nitric acid and the neutralization process by cations (Ca^2+^ and Mg^2+^)^43^. Correspondingly, the input of cations and anions in rainwater was 48.22 and 51.78% during the study period. The outcome shows that the higher contributions of anions are due to transference from distant sources instead of localized sources, which agrees with the other research^25^. Calcium (Ca^2+^) was observed as the dominant ionic species with a much lower influence of SO_4_^2−^ and NO_3_^−^ (13.62 and 11.81%).

Similarly, the sea salts such as Na^+^ contribute 16.39%, and Cl ^−^ contributes 17.72%, indicating transportation from distant sources and influenced by the sea. Notably, SO_4_^2−^ was the second abundant species, and K^+^ was the least abundant species among the anions during the study period. Figure S3 shows the percentage contribution of measured ionic species in the rainwater of Shaune Garang catchment. The result shows that the maximum contribution in the ionic concentration in the rainwater is Ca^2+^ (22.35%) and the minimum from K^+^ (4.08%) might be due to considerable contribution from marine sources of NaCl (sea salt)^44^ and the crustal source in the form of Ca^2+^ and Mg^2+^. In addition, the contribution from anthropogenic sources SO_4_^2−^ (13.62%), NO_3_^−^ (11.81%), and K^+^ (4.08%) is also observed in rainwater composition, which might be due to the local wood-burning practices for the domestic need^45^. Though a significant alternative source of the perceived ionic composition in the rainwater might be dust and sea salt transported from other areas, the highest VWA ionic species concentration in the rainwater was Ca^2+^, Cl^+^, Na^+^, SO_4_^2−^, and Mg^2+^. Volume weighted mean pH was detected at 4.59 and reached as high as 6.73 with increased Ca^2+^ (56.23 μeq/l) in rainwater in the catchment suggests that Ca^2+^ be the primary neutralizing agent in rainwater, though Mg^2+^ and K^+^ can defuse acidity produced due to SO_4_^2−^ and NO_3_^−^ to regulate the pH of rainwater in the alkaline range.

### The ionic ratio of rainwater

Rainwater quality measures a characteristic function such as acidic and alkaline ingredients. The present research found that the ionic strength and concentration in the rainwater during the study period were calculated as 169.14 μeq/l. It is established that the acidic nature of water is generally due to the influence of nitric and sulfuric acid and the neutralization process by cations containing Ca^2+^ + Mg^2+^ ^21,24^. However, the ionic ratio has been calculated to apprehend the comparative involvement of sulfuric and nitric acid rain formation in the study area. Also, the fractional acidity (FA) was calculated to understand the acid neutralization capacity of rainwater in the catchment. The following equation evaluated the correlation between acidic and neutralizing species^46^.

3 $${\text{FA}}._{{{\text{xi}}}} = \frac{{\left[ {{\text{H}}^{ + } } \right]}}{{\left[ {{\text{SO}}_{4}^{2 - } + {\text{ NO}}^{3 - } } \right]}}$$

**Figure 5:**
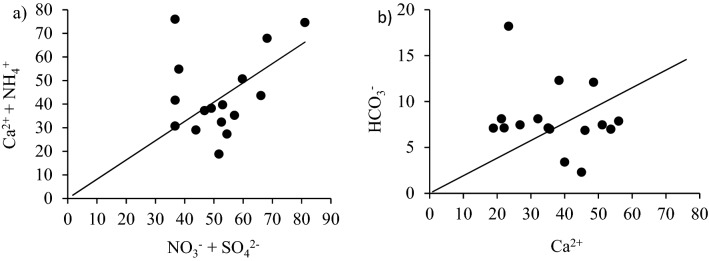
(**a**) Scatter plot of (Ca^2+^  + NH_4_^+^) against (NO_3_^−^ + SO_4_^2−^) and (**b**) Ca^2+^ against HCO_3_^−^ during the study period in Shaune Garang catchment.

The average H^+^/ (NO3^−^ + SO_4_^2−^) ratio is measured as 0.07, indicating the neutralization of 93% of the rainwater. According to the study conducted in the Kothi, North-Western Himalaya reported that almost 96% of rainwater was neutralized^21,24^. The result indicates that rainwater is less acidic in Western Himalayas, particularly in the Shaune Garang catchment, than in North-Western Himalayas. In the urban location, the average H^+^/ (NO_3_^−^ + SO_4_^2−^) ratio of Pune and Delhi was reported as 0.02 and 0.08, demonstrating the neutralization efficiency of 98% and 92%, respectively, by defused alkaline species^47^. The average ratio of (NO_3_^−^ + Cl^−^)/(SO_4_^2−^) was measured as 2.38, indicating a higher value than in North-Western Himalaya, mainly due to the insignificant amount of nitric and hydrochloric acid in rainfall. However, in the case of North-Western Himalaya, the ratio is slightly lower because of sulfuric acid^24^. The equal ratio of NO_3_^−^/SO_4_^2−^ was measured as 0.97 ± 0.60, which suggests the contribution to the acidity in rainwater is dominated by nitric acid and sulfuric acid. The equal ratio of (Ca^2+^  + NH_4_^+^)/(NO_3_^−^ + SO_4_^2−^) is mainly used to evaluate the extent of human activity in water chemistry. Globally, agriculture, including animal husbandry, is the major emitter of ammonia^48,49^, and about 50% of the total global NH_3_ emissions are contributed from Asia^50^. Also, nitrogen-containing gases from the nitrogen-based fertilizer and biomass burning could lead to emission and the formation of NH_3_ in the atmosphere, which gets converted to NH_4_^+^ by gas to particle conversion. All the ionic ratios of the observed ionic concentration in the rainwater discussed in this section for the Shaune Garang catchment are summarized in Table S2. The scatter plot (Fig. 5a) between (Ca^2+^  + NH_4_^+^) against (NO_3_^−^ + SO_4_^2−^) shows the positive correlation of all data set throughout the study period and linear spread beyond the 1:1 equiline with a ratio ranging from 0.48 to 2.73 with the average equivalent value of 1.31 ± 0.51. The result indicates that NH_4_^+^ and Ca^2+^ ions are vital factors in neutralizing acidity in rainwater through CaSO_4_ and (NH_4_)_2_SO_4_. The scatter plot between Ca^2+^ and HCO_3_^−^ has been shown in Fig. 5b. It is imperative from the figures that there is a large variation between Ca^2+^ and HCO_3_^−^. In rainwater, the ionic concentration of Ca^2+^ is much greater than HCO_3_^−^ during the study. The ratio of NH_4_^+^/NO_3_^−^ ranged from 0.42 to 1.00 (mean = 0.81 ± 0.21), while NH_4_^+^/SO_4_^2−^ ranged between 0.21 and 1.46 (mean = 0.73 ± 0.38), which signify the dominancy of NH_4_NO_3_ over (NH_4_)_2_SO_4_ in the atmosphere^51^. Several studies revealed that significant amounts of NH_4_^+^ are released by anthropogenic and natural sources^52,53^. Additionally, the ratios of [NH_4_^+^]/[SO_4_^2−^  + NO_3_^−^] were close to one, suggesting that SO_4_^2−^ and NO_3_^−^ were completely neutralized by NH_3_. Dominance of SO_4_^2−^ was in the form of (NH_4_)_2_SO_4_^2−^ rather than NH_4_H SO_4_^2−^. The amount of SO_4_^2−^ can be estimated by multiplying SO_4_^2−^mass concentration by 1.38. The amount of NO_3_^−^ can be estimated by multiplying the amount of NO_3_^−^ by a factor of 1.29. Furthermore, the result from the ionic composition illustrates that ammonium nitrate is leading over ammonium sulfate composites in the Shaune Garang catchment during the study period, which agrees with other's results^54^.

### Neutralization potential

The acidic and neutralization potential is a significant parameter of understanding rainwater's chemical behaviour. The difference between rainwater's acid and neutralization potential describes the responsible factors for the entire mechanism in the process. Acid potential (AP) is the summation of Nitrate (NO_3_^−^ and non-sea salt (NSS) sulfate (SO_4_^2−^) concentration, and neutralization potential (NP) is the sum of Ca^2+^, Mg^2+^, and K^+^ in the rainwater. The acid potential (AP) and neutralization potential (NP) ratio describe the importance of such factors within the system. The ratio of AP/NP measured as 0.74 in the rainwater during the study period indicates the dominance of neutralization potential. In addition, the neutralizing factors (NF) can also estimate the atmospheric neutralization potential of the chemical constituents in rainwater samples. It measures how well acidic components are neutralized by crustal elements and ammonium ions. Alkaline particles serve a crucial function in modulating the acidity of RW during wet deposition^55^. The two main neutralizing sulfuric and nitric acid agents are calcium and ammonium. The primary supply of calcium is soil dust, whereas the primary source of ammonium is combustion activities. By computing neutralization factors, the potential of Ca^2+^ and Mg^2+^ in neutralization has been established^56^. Neutralization factors can evaluate the ability of crucial alkaline ions in rainfall to be neutralized. The following equation is used to compute the neutralization factors for ions.

4 $${\text{NF}}_{{\left( {\text{x}} \right)}} = \frac{{\left[ {\text{x}} \right]}}{{{\text{NO}}_{3}^{ - } + {\text{SO}}_{4}^{{2^{ - } }} }}$$

where [x] is the concentration of the major ions (Ca^2+^, Na^+^, K^+^, Mg^2+^_,_ and NH_4_^+^) expressed in μeq/L.

The neutralization factor as dominant cations like Ca^2+^, Na^+^, K^+^, Mg^2+^, and NH_4_^+^ in the rainwater of Shaune Garang catchment was measured as 0.85, 0.63, 0.15, 0.34, and 0.39, respectively. The results suggest that the calcium ion (Ca^2+^) in rainwater is the primary controlling neutralizing agent, while potassium is minimum. The high particulate matter (Ca^2+^, carbonates or bicarbonates) in the rainwater buffers the acidity of cloud water, which is widespread in India^57,58^. These findings reveal that Ca^2+^ and Na^2+^ ions, together with NH_4_^+^ ions, are the primary neutralization components with the minimal role of K^+^ in the rain of Shaune Garang catchment. Table S3 shows similar observations on rainwater neutralizing factors in other parts of India. The maximum neutralizing capability in rainwater was calcium (Ca^2+^) and ammonium ions (NH_4_^+^) in different parts of India.

### Contribution of sea-salt and non-sea-salt

An effort was made to study the contribution of various ions in rainwater from sea salt (SS) and non-sea salt (NSS). The input of sea salt and non-sea salt to essential ions in rainwater was assessed by relating the Cl^−^/Na^+^ ratio to seawater (Table S4). On the other side, the input of NSS was calculated by subtracting SS from the total measured ion. The measured ratio of Cl^−^/Na^+^ (1.14) was less than the observed seawater ratio (1.16), showing that sources of sea salt considerably impact the Cl in rainwater in the Shaune Garang area. Furthermore, increased ratios of K^+^/Na^+^, Mg^2+^/Na^+^, Ca^2+^/Na^+^, and SO_4_^2−^/Na^+^ suggest the potential input of additional sources such as soil and nonmarine. The high SO_4_^2−^ values compared to the seawater ratio in the catchment indicate a significant anthropogenic influence. Based on the relative ratios of the major ions, the probable compound formations include NaCl, CaSO_4_, MgSO_4_, MgCl_2_, HNO_3_, NH_4_SO_4_, and (NH_4_)_2_SO_4_^20–26^. The predominant ionic species in rainwater indicate the proportional effect of natural and anthropogenic sources. Natural sources include alkaline and insoluble components of mineral aerosols from the earth's crust and sea. The influences from volcanic sources are insignificant at the sample site. The enrichment factor (EF) is calculated using Na^+^ as the reference element.

5 $${\text{EF}}_{{\left( {{\text{Xi}}} \right)}} = { }\frac{{\frac{{{\text{Xi}}}}{{{\text{Na }}\left( {{\text{rainwater}}} \right)}}}}{{\frac{{{\text{Xi}}}}{{{\text{Na }}\left( {{\text{seawater}}} \right)}}}}$$

where Xi is the required significant ion, Xi/Na (rainwater) is the rainwater ratio, and Xi/Na (seawater) is the seawater ratio.

The EF unity infers no enrichment and, as a result, no input from any source other than seawater. On the other hand, EF value greater than one indicates enrichment of a specific ion relative to non-sea salt sources. It was discovered that the EF of all significant ions (Mg^2+^, K^+^, Ca^2+^, and SO_4_^2−^) was more than one, specifying an essential input from nonmarine sources in the catchment. The result shows that the enrichment value of Cl^−^ is less than 1 indicating marine influence. SSF fraction of 98.27% and NSSF fraction of 1.73% further clarify the influence of marine and nonmarine sources, respectively (Table S4). The enrichment factor of Shaune Garang, compared with the other studies in India, indicates that the EF value of Kothi, Himachal Pradesh (Table S5) is very similar to the present study. It verifies the dominance of nonmarine contributions in the Shaune Garang catchment and even the locations of Himachal Pradesh. However, our observation, compared with the previous research from Nainital, Uttarakhand, indicates that the chemical configuration of rainwater in the Himalayan region is affected by both marine and nonmarine sources.

### Air mass trajectory analysis

Particulate matter concentrations have risen rapidly in developing countries such as India because of high emissions from various human-induced activities^59,60^. The researchers studied the influence of aerosols on the earth's radiation budget, weather parameters like rainfall, and cloud formation^61,62^. Aerosol emissions are to be blamed for rising regional temperatures and have been identified as a critical factor in melting the Himalayan glaciers^63^. The Himalayan region has also been affected by heating caused by another amalgamation of solar energy due to aerosol brown clouds^64^. It can similarly decrease the albedo of snow and glaciers, leading to enhanced melting because of the accumulation of radiation-absorbing aerosols on snow and glaciers. The earlier study^65^ has estimated that the annual average melting of the Himalayas reached 0.7–0.85 m w.e. per annum at Lahaul/Spiti glaciers from 1999 to 2004.

**Figure 6:**
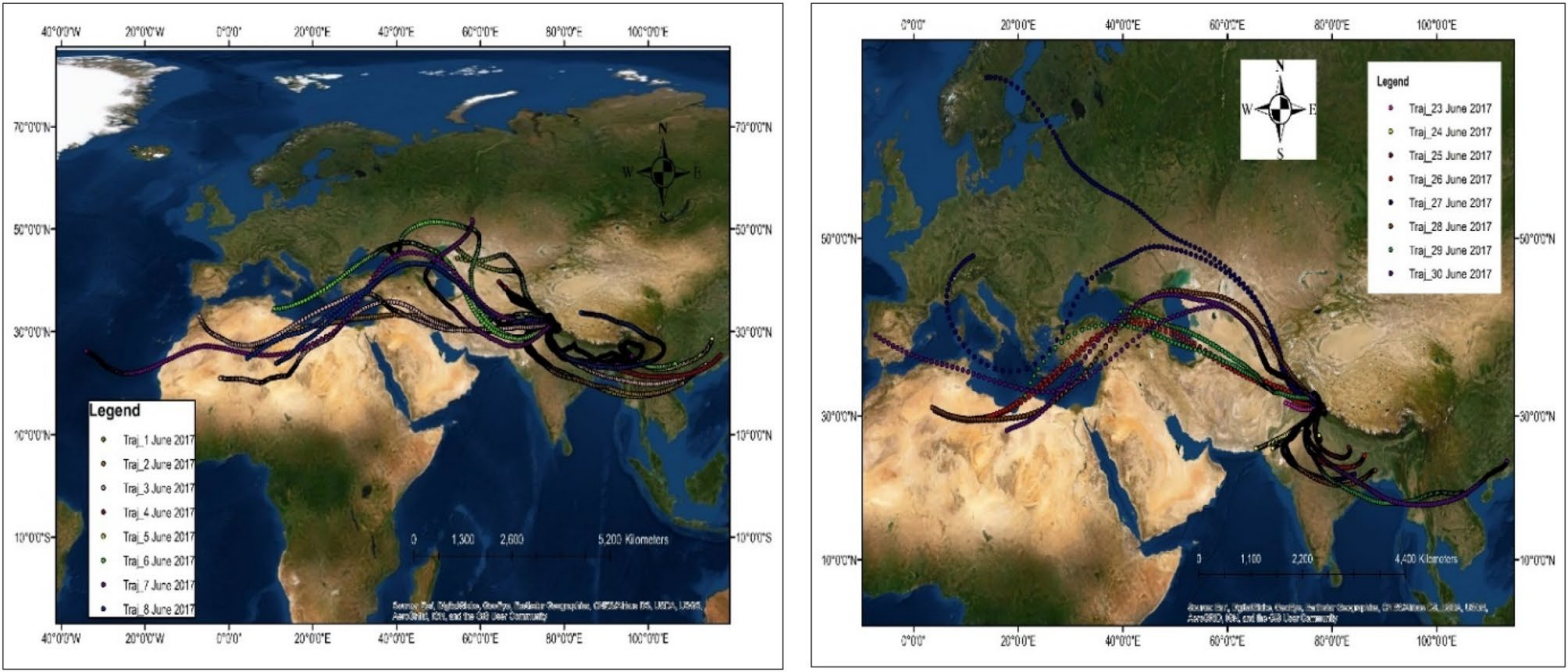
Backward trajectories at rainwater sampling site in Shaune Garang glacier catchment. The NOAA Air Resources Laboratory's Hybrid Single-Particle Lagrangian Integrated Trajectory (HYSPLIT) model (http://www.arl.noaa.gov/ready/hysplit4.html) was used to access the origin of air parcels reaching to Shaune Garang catchment at an altitude of 1000–1500 m above the glacier surface. The READY model (Real-time Environmental Applications and Display System) allows users to access and display meteorological data products and run the HYSPLIT transport and dispersion model on the NOAA Air Resources Laboratory's (ARL) web server.

Moreover, the backward air trajectory modelling significantly showed the impacts up to the Mt. Everest region due to high aerosol emissions from the North-west region. Anthropogenic activities can influence the rainwater's characteristics due to the presence of aerosol^66^. The air mass back trajectories are critical for determining the potential transportation paths of air mass to the station and have been computed at the study area using the NOAA Hybrid Single-Particle Lagrangian Integrated Trajectory (HYSPLIT) model^67^. GDAS (Global Data Assimilation System) data were used to generate three-dimensional back trajectories^68^ for the monitoring location in Shaune Garang glacier on precipitation days. During June, 60% of air parcels were coming from the northwest direction at relatively higher levels (1000–1500) which carry dust particles from the Thar Desert/ Arabia Peninsula and fossil fuel emissions from northwest-west countries (Afghanistan, Pakistan and Iran) to the study locations (Fig. 6). The speed of the wind and the atmospheric boundary layer's height plays an essential role in the diurnal variability of pollutant concentrations and dispersion. The measured ion concentration was approximately two and a half times higher than the air masses passing from the southwest. According to the research, the bulk air masses arrive from the west (the Mediterranean Sea or the mid-west Atlantic Ocean), known as western disturbances. These air masses pass over the Persian Gulf, Iran, Afghanistan, and Pakistan, bringing torrential rainfall to the western Himalayas. Long-distance dust movement interacts with anthropogenic emissions along the dust track, raising local particulate matter concentrations^69^. It was observed that the measured ion concentration was approximately two and a half times higher when the air masses sources were from the northwest.

### Chemometric analysis

The correlation coefficient matrix was made among the measured chemical components in rainwater to identify the relationship between all chemical species (Table S6). The correlation analysis specifies chemical parameters such as SO_4_^2−^– NO_3_^–^, Mg^2+^– NO_3_^–^, Na^+^–SO_4_^2−^, Ca^2+^– Cl^−^, SO_4_^2−^–Cl^−^ and NO_3_^–^ –HCO_3_^–^ have a reasonable correlation. The statistical benchmark is considered significantly more than 0.50 for the correlation analysis in this study. The correlation coefficient (0.50) between Cl^−^ and SO_4_^2−^ indicates the influence of crustal soil. In addition, a minimal correlation (0.22) between bicarbonate and soil-derived Ca^2+^ was observed among the ions, indicating that the contribution of these sources was influenced by soil dust containing large fractions of CaCO_3_ originating from the same sources. The correlation between Ca^2+^ and NO_3_^−^ was observed to be 0.44 during the study period, suggesting that soil is the most important source of nitrate in rainwater, which is present in the atmosphere in the form of Ca (NO_3_)_2_^24^. Minor contaminants like sulfate (SO_4_^2−^) and nitrate (NO_3_^−^) are significantly correlated (0.67), showing their origin from the same sources. In the catchment, the controlling contribution factor of SO_4_^2−^ and NO_3_^−^ in acid formation was nearly 54.64% and 45.38%, respectively. Therefore, the findings demonstrate that the high concentrations in the Himalayan area are thought to be caused by airborne primary and secondary particle movement. A good correlation (0.56) observed between Na^+^ and Cl^−^ suggests that most Na^+^ and Cl^−^ components originated from marine sources and were transported with air masses. However, the ionic ratio of Cl^−^/Na^+^ in the rainwater compared to the seawater ratio in the previous section (Table S4) indicates a part of chloride from other emission sources.

Factors and principal components (PCs) have been analyzed to understand potential sources of ionic species in rainwater. The Bartlett's sphericity test was applied to the data of rainwater, and a correlation matrix was prepared to show χ2 (observed) = 84.93, which is considerably larger than the critical value of χ2 = 30.61 (df- 45 and *p*-value < 0.0001, significance level 0.05). The Pearson correlation coefficient between the measured chemical components in rainwater supported the factor components. In principal component analysis, the data were exposed to a varimax rotation, which optimizes the variance to generate a pattern of loading on each factor as different as possible, allowing for a more straightforward interpretation. Factor loading represents the correlations of each variable with the factor. In this study, four factors were identified, and each factor was assigned a loading. When the loading of each variable was determined to be greater than 0.50, the significant loading was considered. Table 1 summarizes the loading with the variance extracted by the factors (Eigenvalue).

**Table 1:** Principal components (PCs) loading for selected physicochemical parameters in rainwater.

	F1	F2	F3	F4
pH	− 0.672	0.306	0.148	− 0.069
EC	0.276	0.648	− 0.296	− 0.458
Na^+^	0.299	0.763	0.101	− 0.078
K^+^	− 0.072	− 0.545	0.699	0.079
Ca^2+^	0.771	− 0.230	0.277	− 0.072
Mg^2+^	− 0.541	0.452	− 0.059	0.623
Cl^−^	0.596	0.362	0.389	0.427
SO_4_^2−^	0.423	0.646	0.480	− 0.121
NO_3_^−^	− 0.685	0.534	0.250	0.007
HCO_3_^−^	− 0.643	− 0.091	0.480	− 0.470
Eigenvalue	2.924	2.492	1.363	1.037
Variability (%)	27.894	24.981	14.640	10.857
Cumulative (%)	27.894	52.875	67.515	78.373

**Figure 7:**
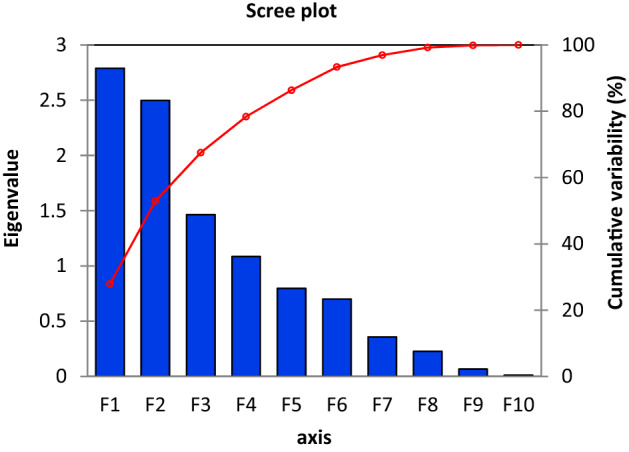
Scree plot of eigenvalue and cumulative variability against the Principal Component Factors.

The analysis resulted in rotated principal components (PCs), Eigenvalues, percentage of variation, and cumulative percent variability (Table 1)**.** All the principal components (PCs) with their Eigenvalues are considered more than 1 to assess the governing factor in the rainwater. The factor extraction were performed for the Eigenvalues greater than 1 after varimax rotation (Moller et al. 2005). The result of factor loading shows that four factors with Eigenvalues greater than 1 explain about 78.37% of the total variance. Scree plot (Fig. 7) of eigenvalue and cumulative variability against the Principal Component Factors measured the cumulative variability as 78.37%, including four-component (PC1 explained 27.89%, PC2 explained 24.98%, PC3 explained 14.64%, PC4 explained 10.85%). However, Factor 1 explains 27.89% of the data variance and displays a robust optimistic loading for Ca^2+^ and Cl^−^. High loading of Ca^2+^ and moderate loading of SO_4_^2−^ signify that the most important sources of these ions are burning fossil fuel and soil dust.

In addition, the first two PC loading interprets the grouping and association amongst the variables. Results indicate the importance of factors I and II, which explain 52.87% of the total variance (Fig. S4a,b). High loading of a chemical constituent such as Cl^–^, Ca^2+^, Mg^2+^, NO_3_^–^ and HCO_3_^−^ demonstrates the influence of natural sources like sea, and soil. Factor II comprises NO_3_^–^, K^+^, and SO_4_^2−^, indicating that the origins of these chemical constituents are vehicular emissions and biomass burning. Although Na^+^ and Cl^−^ generally come in the form of sea salt but give the impression of the same factor. Apart from this, most of the Mg^2+^ shows the origin of the sea source. The SO_4_^2−^ over the study region is primarily due to inadequate fuel burning, fertilizer uses, thermal power plants, refineries, and long-range transport.

### Comparison of ion concentrations in rainwater at higher altitude regions

The ionic concentration of the rainwater in the Shaune Garang catchment was compared with the ionic concentration of rainwater in Himalayan locations such as China, Nepal, Tibet, and India (Table 2). However, it is imperative to emphasize that the most relevant parameters accelerating acid rain formation are NO_3_^−^ and SO_4_^2−^. In the Shaune Garang catchment, high intensities of SO_4_^2−^ (23.83 μeq/l) and NO_3_^−^ (18.75 μeq/l) were observed during the study period. Therefore, these two parameters are the primary inorganic ions formed from SO_4_^2−^and NO_3_^−^ gases during precipitation, emitted from different sources such as vehicular emissions at high altitudes^70^. The concentration of NO_3_^−^ in the sampling area (18.75 μeq/l) surpassed the concentrations observed in the study area of Kathmandu (12.75 μeq/l), Dhunche (10.70 μeq/l), Dimsa (8.52 μeq/l), Gosainkunda (4.40 μeq/l), Southern Everest region (0.01 μeq/l), Northern Everest region (1.10 μeq/l), Nam co Tibet (10.30 μeq/l), Lhasa (2.0 μeq/l), Southern Tibet plateau (2.33 μeq/l), Nainital, Uttrakhand (11.9 μeq/l). Therefore, with the known fact that NO_3_^−^ is a pioneering gas for forming acid rain, it produces nitric acid causing high nitrification with the metal's mobilization in the soil. Consequently, SO_4_^2−^ originates primarily from vehicular and industrial effluence and is the source of acid rain with the presence of sulfuric acid. The concentration of Na^+^ (27.66 μeq/l) and Cl^−^ (31.28μeq/l) was lower in Shaune Garang (Himachal Pradesh) in comparison to Nainital (Uttarakhand), showing Na^+^ and Cl^−^ as (49.8 μeq/l) and (67.3 μeq/l) respectively. According to the European Union, Air Pollution Study Group recommends that the Cl^−^/Na^+^ ratio between 0.5 and 1.5 indicate a marine source. The Cl^−^/Na^+^ ratio of 1.14 of Shaune Garang suggests a marine origin. The values are within the range, and it is possible that the Na^+^ also might have a marine origin. The concentration of HCO_3_^−^ (8.09 μeq/l) in the Shaune Garang catchment is lower than the reported values of other Himalayan locations and may only be influenced by the local origin of soil and limestone^71,72^. Apart from these parameters, Mg^2+^ (13.98 μeq/l) is observed to be high compared to other Himalayan locations, but K^+^ (6.56 μeq/l) and Ca^2+^ (37.13 μeq/l) are almost similar. These ions (Mg^2+^, Ca^2+^, and K^+^) primarily originate from seawater, soil, forest fires^73^ and agricultural sector^74^.

**Table 2:** The average chemical constituent of rainwater from Shaune Garang catchment and its comparison with a chemical constituent of rainwater from other high-altitude Himalayas (unit in µeq/l).

Study site	Cl^−^	NO_3_^−^	SO_4_^2−^	Na^+^	K^+^	Mg^2+^	Ca^2+^	HCO_3_^−^	References
Shaune Garang	31.28	18.75	23.83	27.66	6.56	13.98	37.13	8.09	Present study
Kathmandu	5.18	12.75	16.27	3.80	2.48	4.56	39.15	63.40	^75^
Dhunche	4.54	10.70	9.58	3.69	2.18	3.33	24.61	36.00	^75^
Dimsa	22.73	8.52	7.57	21.13	3.74	5.78	18.64	33.10	^75^
Gosainkunda	14.97	4.40	6.91	10.83	3.93	2.50	9.50	21.40	^75^
Southern Everest region	0.45	0.01	0.06	0.51	0.15	0.05	1.10		^76^
Northern Everest region	1.00	1.10	0.70	0.40	0.20	0.20	1.70		^77^
Nam co Tibet	19.10	10.30	15.50	15.40	14.40	4.70	65.50	72.30	^78^
Lhasa	21.70	2.00	2.50	89.00	14.80	5.70	150.30	288.90	^79^
Southern Tibet Platue	6.74	2.33	2.62	6.73	1.88	1.66	34.40		^80^
Kothi	26.00	17.00	25.00	21.00	5.00	10.00	30.00	4.4	^24^
Nainital	67.30	11.90	18.60	49.80	39.10	43.30	127.40	8	^26^

## Conclusion

The study is primarily concerned with determining the presence of significant ionic concentrations in rainwater from the Shaune Garang catchment. As discussed in the result section, it is influenced by anthropogenic and natural chemical constituents. The volume-weighted mean (VWM) pH value of rainwater ranged between 4.59 and 6.73, with an average value of 5.47 ± 0.69, indicating the alkaline nature of rainfall. The total ionic strength in the rainwater ranged from 113.4 to 263.3 µeq/l with an average value of 169.1 ± 40.4 µeq/l. Total cations and anions during the study were observed 169.4 ± 40.1 µeq/l, and the abundance of ions show a trend as C1^−^  > SO_4_^2−^  > NO_3_^−^  > HCO_3_^−^ for anions and Ca^2+^  > Na^+^  > Mg^2+^  > K^+^ for cations in the rainwater. The major dominant cations were Ca^2+^ (43.1%) and Na^+^ (32%) and anions were Cl^−^ (37.7%), SO_4_^2−^ (28.7%) and NO_3_^−^ (23.8%) in rainwater. The ionic ratios were calculated among all the ions. The fraction of (NO_3_^−^  + Cl^−^) with SO_4_^2−^ was measured as 2.3, which specifies sour faces of rainwater due to HNO_3_, H_2_SO_4_, and HCl. The average ratio of acidic species (SO_4_^2−^/NO_3_^−^) was measured as 1.20, suggesting the higher contribution of SO_4_^2−^ in precipitation. Neutralization factors of 0.87 and 0.32 for Ca^2+^ and Mg^2+^ help neutralize the rainwater. A particular focus has been made on the multivariate statistical assessment of rainwater chemistry. PCA analysis shows the significance of four factors controlling 78.37% of the total variance with individual contributions as 27.89%, 24.98%, 14.64%, and 10.85% for PC1, PC2, PC3 and PC4, respectively. PC1 displays a robust high loading for Ca^2+^ and Cl^−^. Air mass trajectory analysis revealed that most of the air masses are coming from the west (the Mediterranean Sea or the mid-west Atlantic Ocean). Long-range dust transport mixes with anthropogenic emissions along the dust track is responsible for enhancing local particulate matter concentrations. It is observed that 60% of the air parcels reaching the Shaune Garang Catchment were coming from the Arabian Sea and Bay of Bengal in June and the rest from the northwest direction of India. A significant amount of reduction in mass concentrations of ions was observed when the source of origin was the Arabian Sea.

Further, high loading of Ca^2+^ and NO_3_^−^ and moderate loading of SO_4_^2−^ signify the contribution of burning fossil fuel and soil dust. Anthropogenic and natural pollutants influence the composition of rainwater in the pristine Himalayas due to long-distance transportation. The air mass trail investigation demonstrates the impact of airborne primary and secondary particle transportation that influences the chemical composition of precipitation. Hence rainwater composition in the pristine Himalayas is exaggerated mainly by anthropogenic and natural sources of other regions due to long-range transference of air masses from the Mediterranean Sea, mid-west Atlantic Ocean, Arabian Sea Bay of Bengal.

## Supplementary Information

Supplementary Information.

## Data Availability

The datasets used and/or analyzed during the current study are available on reasonable request from the corresponding author.
