# Supplementary material for: Chemometric approach to evaluate the chemical behavior of rainwater at high altitude in Shaune Garang catchment, Western Himalaya
Source: Sci Rep. 2022 Jul 27;12:12774. doi: 10.1038/s41598-022-15422-0 (PMC9329433; doi:10.1038/s41598-022-15422-0)
Supplement: Supplementary file 1 — Supplementary Information. [file 41598_2022_15422_MOESM1_ESM.docx]

**Chemometric approach to evaluate the chemical behavior of rainwater at high altitude in Shaune Garang catchment, Western Himalaya**

**Ramesh Kumar^1^, Rajesh Kumar^1*^, Atar Singh^1^, Mohammad Arif^2^, Pankaj Kumar^3^, Anupma Kumari^4^**

*^1^Department of Environmental Science, School of Earth sciences, Central University of Rajasthan, Bandar Seendri, Ajmer, India.*

*^2^National Institute of Urban Affairs, Ministry of Housing and Urban Affairs, India.*

*^3^Integrated Regional Office, Ministry of Environment, Forest & Climate Change (MoEFCC), Govt. of India, Saifabad, Hyderabad, Telangana, 500004, India*

*^4^Environmental Biology Laboratory, Department of Zoology, Patna University, Patna, India.*

***Corresponding Author:** [rajesh.kumar@curaj.ac.in](mailto:rajesh.kumar@curaj.ac.in)

b

a

Figure S1: Diurnal variation of in situ water parameters a) pH, b) Specific conductivity during the study period 2017 in Shaune Garang catchment.

Figure S2: Linear regression analysis between rainfall (mm) and H^+^ (Hydrogen Ion Concentration (µeq/l).


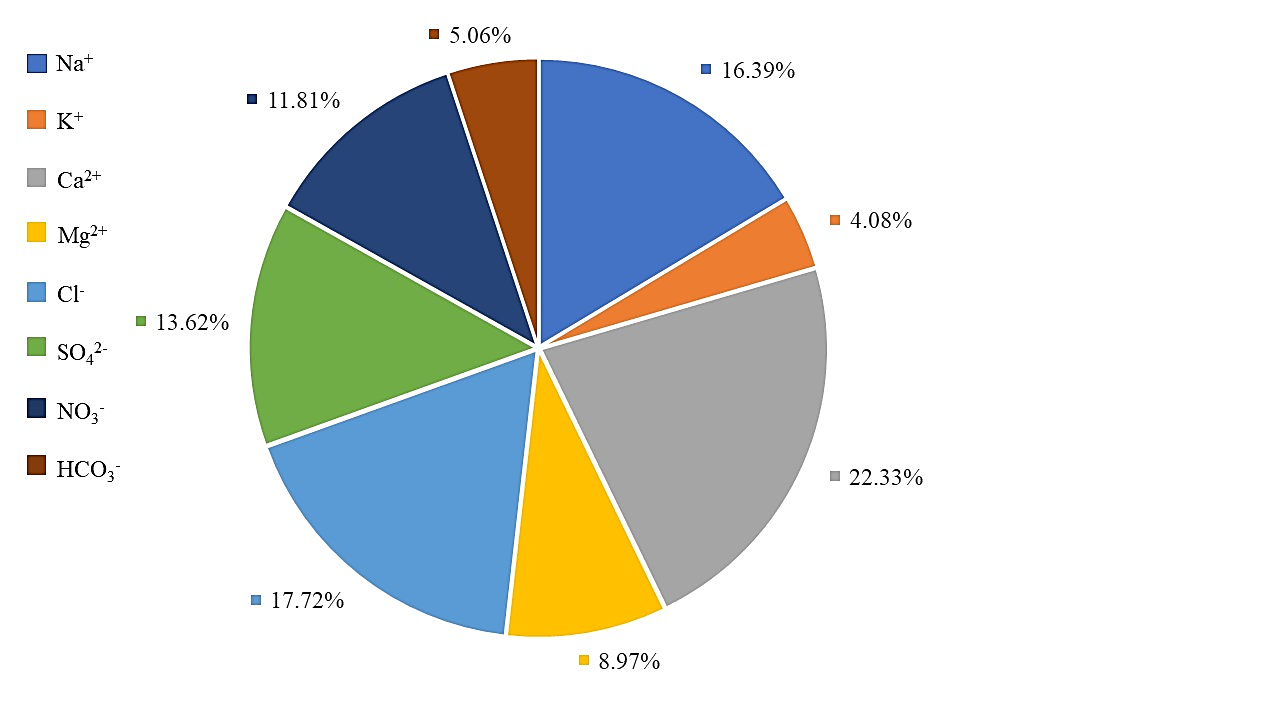


Figure S3: Percentage contribution of measured ionic species in rainwater in Shaune Garang catchment.


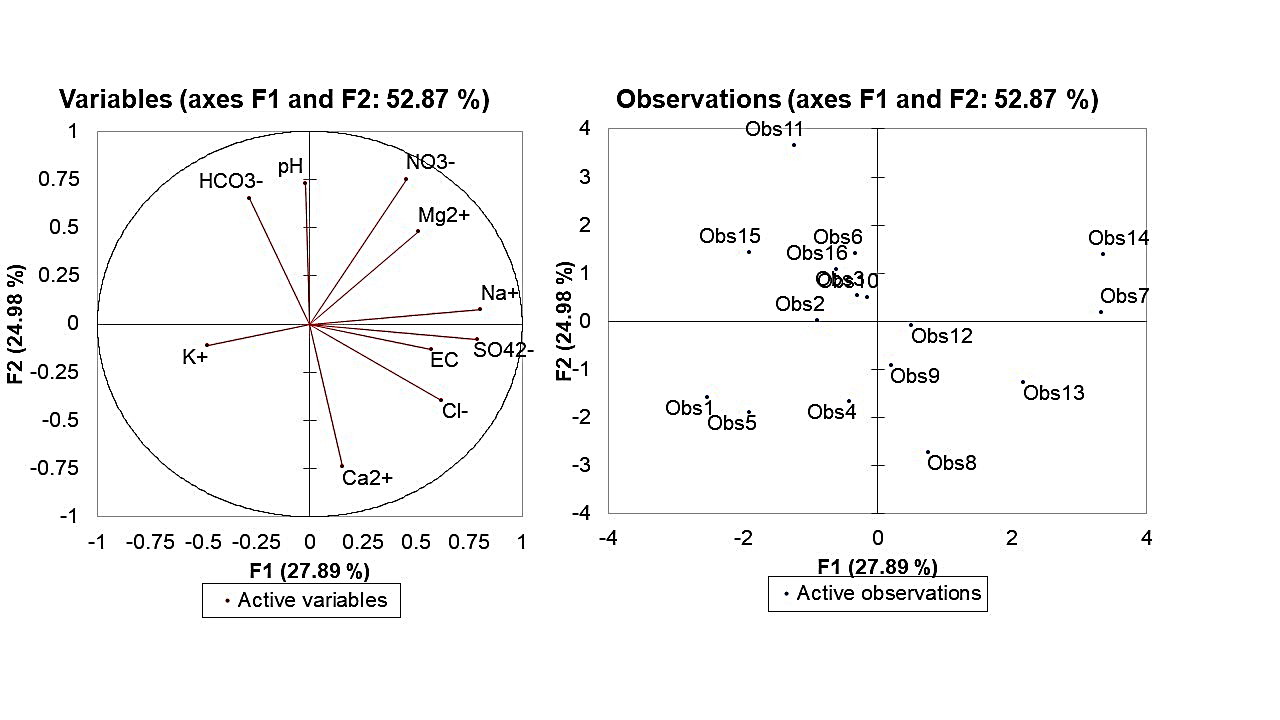


a.

b.

Figure S4: a. Loadings of the chemical constituent of the first two factors. **b.** Score plot of the first two scores.

Table S1. Concentration (μeq/l) of ionic species of rainwater during the summer monsoon season of the year 2017.

| **Parameters** | **Min** | **Max** | **Avg.** | **SD** | **VWM** |
| --- | --- | --- | --- | --- | --- |
| pH | 4.59 | 6.73 | 5.47 | 0.72 | 5.56 |
| Na^+^ | 15.23 | 56.45 | 27.66 | 10.66 | 27.35 |
| K^+^ | 2.31 | 12.98 | 6.56 | 3.25 | 6.86 |
| Ca^2+^ | 18.92 | 56 | 37.13 | 12.23 | 35.7 |
| Mg^2^ | 3.65 | 21.45 | 13.98 | 4.57 | 17.61 |
| Cl^-^ | 11.26 | 56.21 | 31.28 | 16.59 | 29.47 |
| SO_4_^2-^ | 11.51 | 48.29 | 23.83 | 11.98 | 22.32 |
| NO_3_^-^ | 3.65 | 36.17 | 18.75 | 8.75 | 20.61 |
| HCO_3_^-^ | 2.36 | 18.26 | 8.09 | 3.64 | 7.71 |
| NH_4_ | 3.14 | 30.0 | 14.87 | 5.95 | 14.56 |

Table S2: The ionic ratio of the observed ionic concentration in the rainwater from Shaune Garang catchment (unit in µeq/l).

| **Parameters** | **Minimum** | **Maximum** | **Average** | **STD** |
| --- | --- | --- | --- | --- |
| H^+^/ NO_3_^-^ + SO_4_^2-^ | 0.02 | 0.17 | 0.07 | 0.04 |
| NO_3_^-^ / SO_4_^2-^ | 0.24 | 2.47 | 0.97 | 0.60 |
| NH_4_^+^ / NO_3_^-^ | 0.42 | 1.00 | 0.81 | 0.21 |
| NH_4_^+^ / SO_4_^2-^ | 0.21 | 1.46 | 0.73 | 0.38 |
| Ca^2+^ + NH_4_^+^ / NO_3_^-^ + SO_4_^2-^ | 0.48 | 2.75 | 1.34 | 0.51 |
| NO_3_^-^ + Cl^-^ / SO_4_^2-^ | 1.08 | 4.60 | 2.38 | 0.91 |
| NO_3_^-^ + SO_4_^2-^ / Ca^2+^ + Mg^2+^ | 0.36 | 2.07 | 0.87 | 0.39 |

Table S3: Neutralization factors (NF) for selected chemical parameters of Shaune Garang catchment compared with some previous studies in India.

| **Studies** | **K^+^** | **Na^+^** | **Ca^2+^** | **Mg^2+^** | **NH_4_** | **References** |
| --- | --- | --- | --- | --- | --- | --- |
| Shaune Garang, Himachal Pradesh | 0.15 | 0.63 | 0.85 | 0.34 | 0.39 | Present study |
| Kothi, Himachal Pradesh | 0.12 | 0.5 | 0.71 | 0.24 | 0.31 | [1] |
| Manora Peak, Nainital, Uttarakhand | 1.28 | 1.63 | 4.08 | 1.42 | 0.32 | [2] |
| Singhagad, Western Ghat | 0.06 | 0.77 | 0.74 | 0.38 | 0.11 | [3] |
| Mahabaleshwar | 0.13 | 1.45 | 1.86 | 0.65 | 0.24 | [4] |
| Pune, Southwest India | 0.08 | - | 1.4 | 0.29 | 0.29 | [5] |
| Delhi, Northern India | 0.04 | - | 1.4 | 0.49 | 0.17 | [5] |
| Brahmaputra Valley, Tezpur 2012-18 | 0.39 | 0.5 | 1.01 | 0.28 | 1.02 | [6] |

Table S4: Comparison of equivalent concentration ratios of various ionic species concerning sodium ion (Na^+^) along with seawater ratio, sea salt, and non-sea salt (nss) fractions (%) at Shaune Garang catchment.

|  | Cl^-^/ Na^+^ | Mg^2+^/ Na^+^ | K^+^/ Na^+^ | Ca^2+^/ Na^+^ | SO_4_^2-^/ Na^+^ |
| --- | --- | --- | --- | --- | --- |
| Seawater Ratio | 1.16 | 0.22 | 0.02 | 0.04 | 0.12 |
| Shaune Garang | 1.14 | 0.54 | 0.24 | 1.35 | 0.87 |
| SSF | 98.27 | 40.74 | 8.33 | 2.96 | 13.79 |
| NSSF | 1.73 | 59.26 | 91.67 | 97.04 | 86.21 |

Table S5: Enrichment factors (EF) of chemical parameters in rainwater of Shaune Garang catchment and comparison with previous studies.

| **Enrichment Factor** | **Cl^-^** | **Mg^2+^** | **K^+^** | **Ca^2+^** | **SO_4_^2-^** | **References** |
| --- | --- | --- | --- | --- | --- | --- |
| Shaune Garang, Himachal Pradesh | 0.98 | 2.45 | 12.0 | 33.75 | 7.25 | Present Study |
| Kothi, Himachal Pradesh | 1.05 | 1.98 | 10.9 | 32.23 | 9.32 | [1] |
| Monera Park Nainital, Uttarakhand | 1.18 | 3.79 | 38.07 | 61.73 | 3.28 | [2] |
| Panipat, Haryana | 1.08 | 7.84 | 46.79 | 142.6 | 51.12 | [7] |
| NTPC Dadri, Uttar Pradesh | 1.02 | 6.5 | 2.61 | 11.26 | 3.64 | [8] |
| Lucknow, Uttar Pradesh | 1.10 | 17.8 | 1.04 | 106.4 | 12.10 | [9] |
| New Delhi | 1.70 | 1.76 | - | 50.34 | 184.7 | [10] |
| Bhubaneswar, Odisha | 0.9 | 9.08 | - | 5.81 | 4.15 | [11] |
| Sinhagad, Western Ghats | 1.03 | 1.49 | 2.72 | 15.68 | 7.94 | [3] |
| Panipat, Haryana | 1.08 | 7.84 | 46.79 | 142.6 | 51.12 | [7] |
| Rameswaram, Tamil Nadu | 1.07 | 4.14 | 2.61 | 5.44 | 1.61 | [8] |

Table S6: The correlation coefficient among the measured ionic constituents.

| Variables | pH | EC | Na^+^ | K^+^ | Ca^2+^ | Mg^2+^ | Cl^-^ | SO_4_^2-^ | NO_3_^-^ | HCO_3_^-^ |
| --- | --- | --- | --- | --- | --- | --- | --- | --- | --- | --- |
| pH | 1 |  |  |  |  |  |  |  |  |  |
| EC | -0.031 | 1 |  |  |  |  |  |  |  |  |
| Na^+^ | -0.045 | 0.422 | 1 |  |  |  |  |  |  |  |
| K^+^ | -0.084 | **-0.529** | -0.297 | 1 |  |  |  |  |  |  |
| Ca^2+^ | **-0.563** | 0.097 | -0.122 | 0.121 | 1 |  |  |  |  |  |
| Mg^2+^ | 0.295 | -0.090 | 0.072 | -0.224 | -0.481 | 1 |  |  |  |  |
| Cl^-^ | -0.117 | 0.152 | **0.562** | 0.007 | **0.507** | 0.016 | 1 |  |  |  |
| SO_4_^2-^ | 0.006 | 0.365 | **0.688** | -0.068 | 0.275 | -0.009 | **0.502** | 1 |  |  |
| NO_3_^-^ | **0.519** | 0.225 | 0.121 | -0.042 | -0.444 | **0.673** | -0.088 | **0.678** | 1 |  |
| HCO_3_^-^ | 0.405 | -0.206 | -0.181 | 0.278 | -0.220 | 0.045 | -0.410 | -0.068 | **0.536** | 1 |

**References**

1. Tiwari, S., Chate, D. M., Bisht, D. S., Srivastava, M. K., Padmanabhamurty, B. Rainwater chemistry in the Northwestern Himalayan Region, India. *Atmospheric Research* 104-105, 128–138 (2012). <https://doi.org/10.1016/j.atmosres.2011.09.006>.
2. Bisht, D. S. *et al.* Chemical characterization of rainwater at a high-altitude site “Nainital” in the central Himalayas, India. *Environmental Science and Pollution Research* 24(4), 3959–3969 (2017). <https://doi.org/10.1007/s11356-016-8093-z>.
3. Budhavant, K. B., Rao, P. S. P., Safai, P. D., Granat, L., Rodhe, H. Chemical composition of the inorganic fraction of cloud-water at a high altitude station in West India. *Atmospheric Environment* 88, 59–65 (2014). <https://doi.org/10.1016/j.atmosenv.2014.01.039>.
4. Waghmare, V.V., Aslam, M.Y., Yang, L. et al. Inorganic Ionic Composition of Rainwater at a High Altitude Station over the Western Ghats in Peninsular India. *J Atmos Chem* 78, 59–76 (2021). <https://doi.org/10.1007/s10874-021-09416-x>.
5. Rao, P. S. P. *et al*. Sources of chemical species in rainwater during monsoon and non-monsoonal periods over two mega cities in India and dominant source region of secondary aerosols. *Atmospheric Environment* 146, 90–99 (2016). <https://doi.org/10.1016/j.atmosenv.2016.06.069>.
6. Ahmed, M.S., Bhuyan, P., Sarkar, S. et al. Seven-year study of monsoonal rainwater chemistry over the mid-Brahmaputra plain, India: assessment of trends and source regions of soluble ions. Environ Science and Pollution Research (2021). <https://doi.org/10.1007/s11356-021-17385-7>.
7. Tiwari S, Srivastava MK, Bisht DS. Chemical composition of rainwater in Panipat, an industrial city in Haryana. Indian J Radio Space Phys 37:443–449 (2008).
8. Salve, P.R., Gobre, T., Lohkare, H. et al. Source identification and variation in the chemical composition of rainwater at coastal and industrial areas of India. J Atmos Chem 68, 183–198 (2011). <https://doi.org/10.1007/s10874-012-9217-6>.
9. Khare P, Goel A, Patel D, Behari J. Chemical characterization of rainwater at a developing urban habitat of Northern India. Atmospheric Research 69(3–4):135–145 (2004).
10. Kulshrestha UC, Sarkar AK, Srivastava SS, Parashar DC. A study on short-time sampling of individual rain events at New Delhi during monsoon, 1994. *Water Air Soil Pollution* 85(4):2143–2148 (1995).
11. Das N, Das R, Chaudhury GR, Das SN. Chemical composition of precipitation at background level. *Atmospheric Research* 95(1):108–113 (2010).
